# Nicotine-induced changes in surface properties of restorative materials and dental enamel: An in vitro study on flavored e-cigarette exposure

**DOI:** 10.18332/tid/202876

**Published:** 2025-05-16

**Authors:** Maria Salem Ibrahim, Fatimah M. Alatiyyah, Abdulsalam M. Alsalman, Rayan F. Alzenidi, Ali A. Albattat, Ahmed S. Alkhaldi

**Affiliations:** 1 Department of Preventive Dental Sciences, College of Dentistry, Imam Abdulrahman Bin Faisal University, Dammam, Saudi Arabia; 2 College of Dentistry, Imam Abdulrahman Bin Faisal University, Dammam, Saudi Arabia

**Keywords:** resin composite, electronic cigarette, hardness, roughness, color

## Abstract

**INTRODUCTION:**

This study aimed to assess the effects of various nicotine concentrations in flavored electronic cigarettes (e-cigarettes) on the surface roughness, microhardness, and color stability of restorative materials and enamel structure.

**METHODS:**

The study utilized nanohybrid packable resin composite restorations, resin-modified glass ionomer (RMGI), and dental enamel samples (n=10). These samples were exposed to electronic cigarettes with different nicotine concentrations (3, 20, or 50 mg) using a customized chamber connected to a vacuum machine. A total of 3600 puffs were administered through cycles consisting of 9 puffs, with each puff lasting 4 s and an interval of 20 s between puffs, resulting in a total cycle duration of 3 min and 36 s. Before and after the exposure, the samples were tested for microhardness (MH), surface roughness (SR), and color changes (ΔE*). Data were analyzed using one-way and two-way analyses of variance (ANOVA). Multiple comparisons among different groups were conducted using Bonferroni’s multiple comparison test with a p-value level set at 0.05.

**RESULTS:**

The study findings indicate that all samples – enamel, resin composite, and RMGI – experienced significant reductions in MH. However, no significant differences were observed among the enamel groups. Higher nicotine concentrations did not significantly affect the MH in the resin composite and RMGI group, but both showed significant differences compared to the lowest concentration (3 mg) (p<0.05). At 3 mg nicotine, enamel exhibited the highest ratio (%) change (-46.81± 24.68), followed by RMGI (-23.27 ± 6.24). At the highest concentration of 50 mg nicotine, enamel demonstrated a ratio (%) change of -25.46 ± 16.39, whereas RMGI with -75.72 ± 3.46 maintained similar degradation levels to the 20 mg group. SR results revealed that while most enamel and all RMGI samples showed no significant changes after nicotine exposure, all nicotine concentrations significantly increased SR in resin composite (nicotine 3 mg: 76.00 ± 11.90 to 165.46 ± 36.06 nm; p<0.05). Additionally, color change demonstrated that RMGI exhibited the greatest color change after exposure to both 3 mg (ΔE*=9.45 ± 2.30) and 50 mg (ΔE*=10.25 ± 1.53 nicotine concentrations (p<0.05), while enamel and resin composite samples did not show clinically detectable color changes at the 3 mg nicotine concentration. The 20 mg nicotine concentration had the most substantial impact across the groups.

**CONCLUSIONS:**

The higher nicotine concentrations showed a greater effect among all samples in the tested groups. All concentrations of nicotine e-cigarettes (3, 20, and 50 mg) significantly affected the MH of all tested groups. In terms of SR, the only group that did not show a significant increase with all nicotine concentrations is the RMGI. In aesthetic perspective, the lower the concentration of nicotine e-cigarettes, the lower the change in color when compared to higher concentrations.

## INTRODUCTION

The World Health Organization (WHO) tobacco indices report that the most recent estimate of the number of adult tobacco users worldwide is 1.25 billion. Global tobacco consumption rates are continuing to drop, according to statistics from 2022. Approximately 1 in 5 persons globally uses tobacco, up from 1 in 3 in 2000 ^1^. Tobacco use remains one of the leading causes of death and disability, presenting a significant global health crisis^2^. In the course of movements against conventional smoking, electronic nicotine delivery systems (ENDS), commonly known as e-cigarettes or vaping products, have emerged as an alternative method for nicotine delivery^2,3^. It is a new class of products that were introduced to the market in 2007 where these devices aerosolize a liquid that usually contains nicotine, flavorings, and humectants using a battery-powered heating element^4^. These battery-operated devices provide users with a different way to consume nicotine, bypassing some of the harmful effects of conventional tobacco products^4,5^. Since their introduction, e-cigarettes and vapes have experienced a significant surge in popularity and are rapidly growing^4^. The availability of e-cigarettes, their wide range of flavors, and the addictive nature of nicotine, make them attractive to both adult and young populations. E-cigarettes are often marketed as a safer alternative to traditional cigarettes, given their lower production of toxic combustion products^5-7^. However, while e-cigarettes do produce fewer harmful substances compared to cigarettes, they still generate a range of dangerous chemicals^7,8^. Exposure to aerosolized substances can affect the oral and respiratory health of users. As the popularity of e-cigarettes continues to grow, understanding their effects on health becomes increasingly crucial in addressing this modern-day dilemma^6-8^.

The design of e-cigarettes consists of an e-liquid tank, a microcontroller, and a battery. After being wicked, this liquid is aerosolized in a heating coil. The user inhales the aerosol, which normally comprises flavoring compounds, propylene glycol, glycerol, and nicotine^8,9^. Despite the large range of e-liquids available, the three main ingredients – base, nicotine, and flavors – are well recognized. Propylene glycol, glycerol, or a combination of the two in different ratios diluted in purified water is used to make the base^10^. The range of nicotine concentrations is 0 mg/mL to 18 mg/mL, with consumers usually selecting their preferred level of nicotine. It is possible to classify flavors according to tastes or scents (such as bread, drinks, fruits, menthol, etc.). Sugar alcohol, such as ethyl maltol, is used to provide the sweet scent in e-liquids, while sucrose or sucralose is added for the sweet flavor^11^.

Cigarette smoking is known to cause a variety of oral health issues, including tooth loss, periodontitis, gingivitis, epithelial malignancies, and tooth staining^10-13^. While e-cigarettes might be perceived as a safer option, they are not without risks. The nicotine content in e-cigarette liquids varies widely, ranging from 6 to 48 mg/mL, compared to approximately 24 mg of nicotine per pack of traditional cigarettes^10^. Additionally, e-cigarette liquids are not meant to be consumed in one sitting; a single cartridge can provide around 200 puffs, equivalent to one to three packs of cigarettes. The impact of e-cigarettes extends beyond health concerns to dental restorations. Despite advancements in restorative materials like composites and resin-modified glass ionomer cement, which closely mimic the natural appearance of dental enamel and dentin, these materials remain susceptible to staining^12,13^. E-cigarette components, such as carbon monoxide and ammonia, can cause significant discoloration, turning restorations yellow or even black. This staining not only affects the color but also alters the surface texture of restorations. While polishing can reduce surface stains, it may not fully restore the original color^14,15^. As e-cigarettes continue to rise in popularity, it is crucial to recognize both their potential benefits and the risks they pose, particularly concerning oral health and dental aesthetics^16^.

Consequently, major concerns with dental restorations and tooth structure may be the effects of e-cigarette smoke. A few articles researched and quantified the extent of damage caused by e-cigarettes on the surface and color of different dental materials^15-17^. However, there is no universal agreement on how exposure to e-cigarettes affects dental materials and enamel structure. This investigation aimed to assess the effects of different nicotine concentrations in flavored e-cigarettes on the surface roughness, microhardness, and color stability of some dental restorative materials and enamel structures. The null hypothesis states that the e-cigarettes will not have a significantly different effect on the microhardness, surface roughness, and color stability of the tested groups in comparison to the baseline and among each other.

## METHODS

### Study design, study groups, and sample size

This study is an *in vitro* laboratory study. It included two restorative materials, resin composite and resin-modified glass ionomer (RMGI), and human enamel samples. Also, the study utilized three different concentrations of e-cigarettes. The study materials are presented in Table 1. The sample size was calculated based on data from a previous study^15^ that assessed the color of resin composite restorations after e-cigarette exposure and found ΔE* = 0.8512 ± 0.589 in comparison to the control group, which showed ΔE* = 0.487 ± 0.262. Using α=0.05 and power of 80%, the calculated sample size was 10 samples per group.

**Table 1:** Manufacturers and composition of study materials

*Study materials*	*Manufactures*	*Composition*
**E-liquid**	VGOD, LUSH-ICE, XL-Vape, SaltnicLabs Line, Torrance, CA, USA	Propylene glycol (PG), vegetable glycerin (VG), water, flavorings (Cuban cigar infused with vanilla custard) and nicotine (3 mg, 20 mg, 50 mg)
**Resin composite**	Filtek Z3503M ESPE, Minnesota, Minn., USA	Bis-GMA, UDMA, TEGDMA, and bis-EMA(6) resins. The fillers are a combination of 20 nm silica filler, 4–11 nm zirconia filler, and zirconia/silica cluster filler
**Resin-modified glass ionomer (RMGI)**	Photac Fil/PF, Ketac N100/KN, 3M Espe, USA	Methacrylate-modified polyalkenoic acid, nano-sized zirconia/silica 69% by weight water, polymerizable methacrylate monomers and photo-initiators

### Ethical approval

Ethical approval was obtained from the IRB Committee at Imam Abdulrahman bin Faisal University before the start of the study (IRB-2023-02-478).

### Sample preparation

Collected permanent maxillary anterior teeth that are usually thrown away after removal were stored in a 0.01% (w/v) thymol solution with pH 7, until required for research^18^. To prepare these teeth for tests, enamel blocks were precisely cut to 3×3 mm dimensions and a thickness of 2 mm using carborundum discs in a straight handpiece. The enamel blocks were then firmly placed in a resin mold measuring 15 mm in diameter and 4 mm in thickness^18,19^. To achieve a texture of human enamel on the surfaces of the enamel blocks, they were polished using a grinder polisher equipped with a vector power machine (EcoMetTM 30 Semi-Automatic Grinder Polisher, Buehler, IL, USA). This polishing process included the use of discs with silicon carbide grit levels of 320, 600, and 1200 while water was used as a coolant^18-20^. This technique resulted in a surface texture resembling that of natural tooth enamel with a roughness (Ra) value of around 0.05 µm.

### Preparation of resin composite and RMGI restorative materials

Disc-shaped samples of nanohybrid resin composite enamel-shaded A1 (Filtek Z3503M ESPE, Minnesota, Minn., USA) and resin-modified glass ionomer (RMGI) were prepared. To standardize the preparation of resin- (Photac Fil/PF, Ketac N100/KN, 3M Espe, USA), capsulated materials were used to ensure consistent powder-to-liquid ratios and uniform mixing^18-20^. To create these samples, a mold measuring 8 mm in diameter and 2 mm in thickness was filled with the resin composite material, which was covered with clear polyester strips and glass slides on both sides^20^. The resin composite was applied and cured for 20 s, followed by an additional 20 s of light-curing on each side using an LED light device (Satelec Mini LED Curing Light 1250 mW/cm^2^, A-dec Inc., Newberg, OR, USA). After a 24-hour period, the edges of the samples were polished with sandpaper without touching the flat tested surfaces (n=10)^17,20^.

### Assessments

*Microhardness test (MH)*

Enamel samples

The hardness of the enamel samples was measured using a micro-indentation hardness tester with a Knoop indenter (BUEHLER MicroMet 6040 Hardness Tester, Shanghai, China) with baseline measurements taken before the experiment. The samples underwent five indentations at a load of 25 g, with a dwell time of 15 s to determine the hardness value based on the average of these readings^18^. For this study, the enamel samples were polished to replicate clinical conditions, and the 491 KH ± 20% threshold was applied to ensure sample homogeneity and exclude outliers^18,20^. Afterwards, we used e-cigarettes with varying levels of nicotine concentration on the samples and assigned them randomly to groups. The hardness ratio change (%) of each sample was calculated according to the formula:

Ratio change (%) = [(MH after application - MH baseline)/MH baseline]×100

Resin composite and RMGI samples

In the experiment, the Vickers indenter was used to test the microhardness of the resin composite and RMGI samples. Five indentations were made on each sample with a 200 g load and a dwell time of 10 s. The final hardness value was determined by averaging these readings^18,19^. In the research, there were resin samples with a microhardness of 104 KH ± 20%, along with RMGI samples having an average microhardness of 97 KH ± 20%. These samples were then randomly assigned to nicotine concentration groups, and tested once more after the application of e-cigarettes. The hardness ratio change (%) of each sample was calculated according to the above formula.

*Surface roughness (SR)*

Surface roughness was assessed by utilizing a contact profilometer (Contour GT K 3D Optical Microscope, from Bruker in Billerica). The primary metric employed in these assessments was the surface roughness average (Ra) indicative of the peak-to-valley differences from the surface line. This parameter is widely acknowledged as a measure of surface roughness^18^. Before experimenting, for accuracy assessment purposes and as a point of reference for comparisons, measurements were taken beforehand. After using e-cigarettes each sample surface was meticulously cleaned using a tissue followed by drying with paper to remove any remaining particles that might disrupt the outcomes. Subsequently, the surface roughness (Ra) was gauged in three regions on every sample while ensuring that these spots were situated away from the area to prevent any impact from the central zone^18^.

*Color assessment (CA)*

Color evaluations of all samples were conducted using a reflectance spectrophotometer (Color Eye^®^ 7000 A, by X Rite from Carlstadt in New Jersey). The CIE L*a*b color scale was utilized for this purpose. A standard illuminate (known as D65) covering wavelengths ranging from 360 to 740 nm was employed. At each time point, during the assessment process, the three-color coordinates (namely L*, a*, b*) were calculated. Any color variations (denoted as ΔE) were computed based on the formula:

ΔE* = [(ΔL*)^2^ + (Δa*)^2^ + (Δb*)^2^]^1/2^

The average values were contrasted among the materials tested both before and after the application was made.

### E-cigarettes application

**Figure 1:**
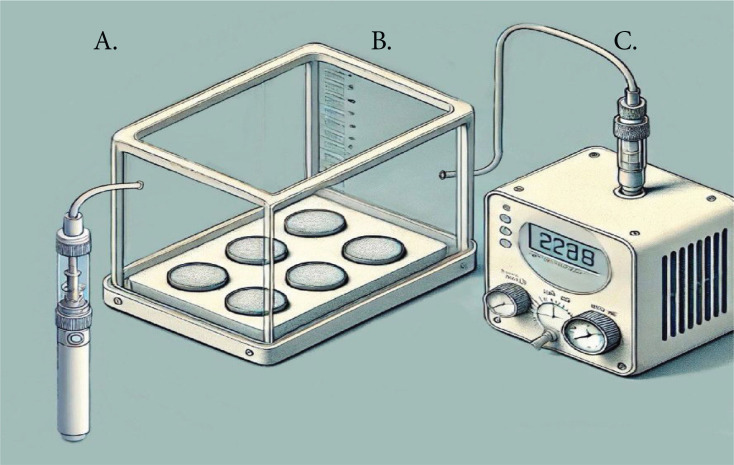
The design for e-cigarette application: A) vapor source of e-cigarettes; B) customized box; and C) vacuum device

The ready samples were randomly split into three sets (each with n=10) representing three varying levels of nicotine content: 3, 20, and 50 mg. All had a flavor profile. The experimental setup used for the application of e-cigarettes comprised a customized container, with sealed openings to regulate the dispersion of vapor effectively throughout the exposure phase. The setup was linked to a vacuum system. Created to replicate conditions in a mouth to keep samples steady while exposing a part of their surfaces to vapor. There were two openings in this setup. One is connected to a vacuum device (Caliburn X POD System by Shenzhen Uwell Technology Co., Ltd. located in Shenzhen city of Guangdong Province in China) that allows regulation of vapor quantity and duration as well as the number of inhalations. The second opening was created for storing the vape tool and refilling the e-fluid VGOD CUBANO SILVER (VGOD brand) used to produce vapor, for research purposes. The selection of this device and e-fluid flavor along with nicotine levels was influenced by their use and accessibility, among consumers. The e-cigarette batteries were charged to capacity. Kept at room temperature consistently during the entire experiment. The vacuum mechanism suct the aerosol produced by the atomizer into the chamber for exposure^15^. By following this configuration (illustrated in Figure 1) a stable and regulated setting was maintained to evaluate how varying nicotine concentrations affected the samples. All experiments were conducted in a laboratory maintained at room temperature (about 22–25°C). Samples were equilibrated to ambient conditions for 24 h before testing, and aerosol exposures were performed sequentially within 2 weeks to minimize environmental variability. While temperature and humidity were not formally controlled, the laboratory environment remained stable throughout the study. All experiments were conducted in a laboratory maintained at room temperature (about 22–25°C). Samples were equilibrated to ambient conditions for 24 h before testing, and aerosol exposures were performed sequentially within 2 weeks to minimize environmental variability. While temperature and humidity were not formally controlled, the laboratory environment remained stable throughout the study.

### Puff cycle and aerosolization method

In this study on vaping habits resembling smoking patterns among users^15^, a total of 180 puffs per dose were carefully chosen to reflect consumption levels similar to those of a pack of cigarettes per day by an average consumer. The experiment involved a total of 3600 puffs administered by an individual who was not aware of which doses were being given out. Each sequence comprised 9 puffs lasting for 4 s, with a gap of 20 s between each puff session, making up a cycle duration lasting 3 min and 36 s for each cycle. As a precaution against issues, fresh e-liquid was supplied after every set of 20 cycles was completed. After administering 180 puffs to each sample, they were softly rinsed with water for 1 min to eliminate any leftover aerosol residue^15^. The vacuum system that sucked out the aerosol from the atomizer operated at a flow rate of 20 mL/min ^17^. This particular flow rate was selected with care to ensure that all aerosol was captured by the trap without needing one. It is worth noting that the e-fluid that was not aerosolized was extracted before the process of aerosolization took place.

### Statistical analysis

The results for MH, SR, and CA are presented as means and standard deviations. The normality of the data was assessed using the Shapiro-Wilk test, and all dependent variables followed a normal distribution (p>0.05). Within-group before-and-after comparisons were performed using paired t-tests, while between-group differences were analyzed using one-way and two-way ANOVA with Bonferroni’s *post hoc* correction for multiple comparisons. Two-way ANOVA was used to assess the interaction between the material type (enamel, resin composite, RMGI) and nicotine concentration (3, 20, and 50 mg) for MH, SR, and CA. All tests were two-tailed with a significance level set at p<0.05. All statistical analyses were performed using Stata/IC 14.2 (StataCorp, College Station, TX 77845) with a p-value level set at 0.05.

## RESULTS

The means and standard deviations obtained in MH are presented in Table 2. The enamel, resin composite, and RMGI samples were all affected by the different nicotine concentrations and showed statistically significant reductions in all groups. However, there were no significant differences in MH in the enamel groups after exposure to different nicotine concentrations. Higher concentrations (20 and 50 mg) showed insignificant differences between after-exposure in the resin composite and RMGI groups. However, both groups showed a statistically significant difference in comparison to the lower concentration (3 mg) (p<0.05) in both the resin composite and RMGI groups. The change in the MH ratio of different nicotine concentrations in the groups is presented in Table 2. At 3 mg nicotine, enamel exhibited the highest ratio (%) loss (-46.81 ± 24.68), followed by RMGI (-23.27 ± 6.24). At the highest concentration of 50 mg nicotine, enamel demonstrated the least ratio value (-25.46 ± 16.39), whereas RMGI (-75.72 ± 3.46) maintained similar degradation levels to the 20 mg group. In the resin composite, the highest ratio change was in the 50 mg concentrations (-78.19 ± 10.70).

**Table 2:** Microhardness (KH for enamel, VH for resin composite, and RMGI)

*Nicotine* *concentration* *(mg)*	*Enamel*			*Resin composite*			*RMGI*		
	*Before*	*After*	*Ratio* *change %*	*Before*	*After*	*Ratio* *change %*	*Before*	*After*	*Ratio* *change %*
3	482.75 ± 75.51 ^aA^	266.61 ± 145.21 ^aB^	-46.81 ± 24.68	111.19 ± 8.75 ^aA^	98.68 ± 6.59 ^aB^	-11.08 ± 4.61	97.31 ± 13.24 ^aA^	72.24 ± 3.94 ^aB^	-23.27 ± 6.24
20	517.62 ± 87.74 ^aA^	301.34 ± 67.51 ^aB^	-41.44 ± 10.46	99.38 ± 11.41 ^aA^	22.06 ± 2.28 ^bB^	-77.64 ± 2.53	97.40 ± 11.69 ^aA^	21.41 ± 1.30 ^bB^	-77.85 ± 1.70
50	538.73 ± 67.77 ^aA^	399.17 ± 87.56 ^aB^	-25.46 ± 16.39	109.71 ± 17.08 ^aA^	24.98 ± 5.41 ^bB^	-78.19 ± 10.70	97.17 ± 11.97 ^aA^	23.30 ± 1.97 ^bB^	-75.72 ± 3.46

Values are given as mean ± standard deviation. Values with different lowercase superscripts in the same column indicate statistically significantly difference (p<0.05) among the different concentrations at one timepoint, while values with different uppercase indicate statistically significantly difference (p<0.05) among the before and after values of the same material (row).

The results for SR are shown in Table 3. Most of the enamel and all RMGI groups did not show any statistically significant change in SR after the application of nicotine. On the other hand, all different nicotine concentrations showed a statistically significant increase in SR in the resin composite samples (p<0.05). Only the lowest concentration (3 mg) of nicotine increased the SR of enamel samples. Enamel showed minimal SR changes in 20 mg: 464.18 ± 124.12 nm to 631.97 ± 245.84 nm; p>0.05, while RMGI remained stable in 50 mg: 289.94 ± 53.47 nm to 330.76 ± 42.43 nm; p>0.05).

**Table 3:** Surface roughness (nm)

*Nicotine* *concentration* *(mg)*	*Enamel*		*Resin composite*		*RMGI*	
	*Before*	*After*	*Before*	*After*	*Before*	*After*
3	365.97 ± 106.83 ^aA^	529.20 ± 112.326 ^aB^	76.00 ± 11.90 ^aA^	165.46 ± 36.06 ^aB^	300.52 ± 44.02 ^aA^	306.60 ± 59.27 ^aA^
20	464.18 ± 124.12 ^aA^	631.97 ± 245.84 ^aA^	89.67 ± 24.63 ^aA^	168.77 ± 24.65 ^aB^	281.14 ± 59.82 ^aA^	295.17 ± 54 ^aA^
50	426.59 ± 159.15 ^aA^	653.77 ± 243.69 ^aA^	87.04 ± 28.8 ^aA^	170.40 ± 60.99 ^aB^	289.94 ± 53.47 ^aA^	330.76 ± 42.43 ^aA^

Values are given as mean ± standard deviation. Values with different lowercase superscripts in the same column indicate statistically significantly difference (p<0.05) among the different concentrations at one timepoint, while values with different uppercase indicate statistically significantly difference (p<0.05) among the before and after values of the same material (row).

Table 4 shows the color change after the application of different nicotine concentrations. Overall, RMGI was the highest (p<0.05) in color change in comparison to enamel and resin composite samples after the 3 mg (9.45 ± 2.30; p<0.05) and 50 mg (10.25 ± 1.53; p<0.05). The enamel samples and resin composite samples did not show clinically detectable changes in color after being exposed to the 3 mg nicotine concentration. The 20 mg nicotine concentration (enamel: 6.96 ± 2.16; resin composite: 9.29 ± 4.16; p<0.05) was the highest in affecting the different groups in comparison to the other two concentrations.

**Table 4:** Color assessment (∆E*)

*Nicotine* *concentration* *(mg)*	*Enamel*	*Resin* *composite*	*RMGI*
3	2.42 ± 2.19 ^aA^	3.35 ± 1.55 ^aA^	9.45 ± 2.30 ^aB^
20	6.96 ± 2.16 ^bA^	9.29 ± 4.16 ^bA^	11.16 ± 2.05 ^aA^
50	5.10 ± 2.34 ^abA^	9.08 ± 3.78 ^bB^	10.25 ± 1.53 ^aB^

Values are given as mean ± standard deviation. Values with different lowercase superscripts in the same column indicate statistically significantly difference (p<0.05) among the different concentrations at one timepoint, while values with different uppercase indicate statistically significantly difference (p<0.05) among the before and after values of the same material (row).

## DISCUSSION

Smoking is a habit that greatly affects both oral and overall health^7-11^. With e-cigarettes being a trend and gaining popularity more than ever before, it is essential to understand their effects better^5,6^. However, the impact of e-cigarette nicotine on teeth and dental materials has not been thoroughly researched. This study aimed to assess how various levels of nicotine in flavored e-cigarettes affect the surface roughness, microhardness, and color stability of materials. Unlike previous studies, this study employed a device that generated smoke from e-cigarettes at a close range over the samples being tested. This method reduces the dispersion of smoke over areas and focuses its effects on the samples^17^. Comparing e-cigarettes to cigarettes poses a challenge in measuring the intensity of e-cigarettes^20,21^. While traditional cigarettes can be estimated based on an intake of 10 to 15 puffs per cigarette smoked each day, this method does not apply to e-cigarette usage patterns, as users often take puffs at various intervals and durations, during different sessions. Collecting data from users regarding their puff counts can be complicated for researchers, due to concerns about accuracy^21,22^. While some devices come with built-in puff counters and manufacturers claim a specific number of puffs per cartridge, accurate information can be limited due to user’s reports and variations in device quality^22^. In contrast to this scenario, the study followed an approach for exposure by ensuring the use of equipment puff counts and exposure durations across different nicotine concentrations. Each session included 200 puffs in line with findings by Robinson et al.^15^. Among 21 cigarette users surveyed by a study of individuals aged ≥15 years, an average of 225 vaping sessions each day was noted. The examination involved a total of 3600 inhalations across trials to simulate smoking five packs of cigarettes over ten days to comprehensively evaluate nicotine staining from e-cigarettes. This controlled approach is critical for providing clearer insights into the effects of e-cigarette use on material characteristics. While existing literature often lacks standardization in exposure methods, this study’s design aims to fill that gap and contribute to a better understanding of the implications of e-cigarette use.

This study demonstrates that nicotine concentration in flavored e-cigarettes significantly impacts the surface properties of dental restorative materials and enamel. All materials exhibited reduced MH after exposure, with resin composite and RMGI showing concentration-dependent declines. SR increased significantly in resin composite across all nicotine levels while enamel and RMGI remained unaffected (p>0.05), and RMGI exhibited the greatest color change, whereas enamel showed clinically detectable discoloration only at 20 mg. These results underscore material-specific vulnerabilities to nicotine exposure. The findings of this research suggest that being exposed to nicotine can cause a significant reduction in MH materials like resin composites and RMGI when the concentration is high, whereas enamel seems to react consistently to nicotine regardless of the concentration levels without any major variations noted among them. Nevertheless, long-term and continuous exposure to enamel may result in degradation even though enamel might exhibit resistance to nicotine exposure compared to dental restorative materials. These findings are consistent with previous studies, which have also reported detrimental effects of smoking and nicotine on enamel hardness but suggest that the damage may plateau at higher nicotine concentrations^23-25^. In terms of resin composite and RMGI materials, it implies that nicotine levels surpassing a threshold could reach a point where higher concentrations beyond a level (for example, 20 mg) do not lead to additional notable deterioration in these substances. The research of Bertold et al.^[Bibr CIT0023]23^ aligns with the observed pattern, indicating that smokers tend to have lower enamel MH levels due to cumulative harm from prolonged nicotine exposure impacting dental structure over time. However, resin composite and RMGI appear to be more responsive to nicotine levels compared to enamel, which showed no changes across concentrations of nicotine. The variations between enamel and restorative materials can be linked to their composition and structure. Enamel is made up of hydroxyapatite, which is a highly mineralized tissue, whereas resin composites and RMGIs are synthetic materials containing organic matrix components^25-27^. Nicotine tends to permeate and interact with the polymer matrix in resin composite and RMGI materials, which may result in a significant decrease in MH levels. Previous studies have emphasized the susceptibility of resinbased materials to elements such as nicotine that can alter their physical characteristics over time^25,26^. Additionally, the results of this study align with those of Rosbrook et al.^27^, which found that the design of devices and the content of e-cigarette liquids, like nicotine, can impact the extent to which the surface deteriorates over time.

The study findings show variations in the SR of materials when exposed to different levels of nicotine concentration. In particular, enamel and RMGI groups did not display any changes in SR after exposure to nicotine. Resin composite samples showed an increase in SR across all nicotine concentrations tested. It is worth noting that only the lowest nicotine concentration (3 mg) led to a rise in SR for enamel samples. The results for resin composites align with studies that found a regression in surface quality of resin composites when exposed to different chemical substances, like nicotine, compared to dental materials such as enamel and RMGI^17,25^. Resin composites can develop surfaces when exposed to nicotine due to its interaction with the materials matrix which leads to surface degradation^25^. For instance, resin composites are prone to surface roughening under the influence of nicotine, likely due to its interaction with the organic matrix of the material, causing degradation of the surface structure. It was observed that there were no differences in surface roughness (SR) for the enamel and RMGI groups when exposed to levels of nicotine (20 and 50 mg). This increase in roughness can affect both the material’s aesthetics and its long-term performance, as rough surfaces are more prone to plaque accumulation and bacterial colonization, potentially compromising the longevity of dental restorations. Enamel is known for its strong structure, and previous research has indicated that it tends to be quite resilient against chemical deterioration^25^. Bertold et al.^[Bibr CIT0023]23^ found no significant changes in the enamel surface roughness of smokers versus non-smokers, indicating that while nicotine may affect enamel hardness, it does not always manifest in significant surface roughening. This implies that although nicotine could potentially impact enamel hardness to an extent, it does not necessarily result in high roughness of the surface.

These study results align with the notion that nicotine’s influence on enamel may be limited specifically to properties such as hardness rather than surface texture. Unlike enamel’s reaction to nicotine concentrations, which were found to vary significantly in this study, RMGI shows surface resistance across all levels of nicotine, indicating its natural ability to resist nicotine-induced degradation. A previous study pointed out that the addition of glass ionomer in RMGIs resin matrix improves the material’s chemical durability^24^. This durability could explain why only minimal changes in surface roughness were observed in RMGI samples compared to resin composites that showed roughening. These findings align with those of Yamamoto et al.^28^ who that RMGI exhibits resistance to wear and surface changes caused by various factors; this makes it a preferred material for applications in environments that are exposed to different chemical substances, such as nicotine. The significant increase in surface roughness (SR) observed in resin composite samples after nicotine exposure carries critical clinical implications. While the post-exposure SR values (≤170.40 nm) remain below the 200 nm (0.2 µm) threshold associated with enhanced bacterial adhesion and biofilm formation^29^, prolonged exposure in clinical settings – combined with organic deposits from saliva or dietary habits – could exacerbate surface degradation, increasing plaque retention and secondary caries risk^30^. Rough surfaces create microretentive niches for cariogenic bacteria like *Streptococcus mutans*, compromising restoration longevity and periodontal health^30^. For resin composites, this underscores the need for frequent polishing or replacement in high-risk patients, such as habitual e-cigarette users. In contrast, RMGI’s stable SR profile (ΔSR <10 nm; p>0.05) aligns with its clinical reputation for plaque resistance^24^, making it a prudent choice for patients with nicotine exposure. These findings highlight the importance of post-restoration polishing protocols and material-specific selection to mitigate long-term biocompatibility risks.

The study findings show that dental materials respond differently to nicotine levels in terms of color stability; RMGI is most sensitive to color alteration at 3 and 50 mg nicotine concentrations. The enamel and resin composite samples did not exhibit color changes following exposure to the 3 mg concentration; however, significant color alterations were observed with the 20 mg nicotine concentration across all groups. The results indicate that the level of nicotine in materials significantly affects their discoloration process and varies depending on the type of material used. RMGI materials are susceptible to staining and discoloration because of their water-attracting properties, which enable them to absorb water and substances like nicotine, resulting in increased color changes; in addition to the point mentioned earlier about the resin part in RMGI being possibly affected more by nicotine’s chemical reactions leading to the color variations. Conversely, enamel and resin mixtures showed color retention at nicotine levels (around 3 mg) with no visible color alterations that could be detected in a clinical setting, consistent with previous research outcomes^29-31^. Resin composites are less likely to change color when exposed to amounts of staining agents due to the properties of the resin matrix and filler particles^29^. Nonetheless, resin composites tend to become more susceptible to discoloration with prolonged exposure to concentrations of staining substances. This was supported by the work of Karanjkar et al.^30^, which revealed that while resin composites maintain color stability at first, their proneness to staining grows when confronted with increased levels of staining agents like nicotine. Enamel is a highly mineralized tissue that is less likely to get stained externally compared to resin-based materials, because of its low porosity and resistance to absorbing outside substances^25^. Enamel is less likely to get stained by nicotine compared to other materials due to its high mineral content. The highest discoloration was observed with a 20 mg nicotine concentration on all tested materials, such as enamel, resin composite, and RMGI^28^. This indicates that nicotine causes more significant staining at higher concentrations, possibly because it can infiltrate and attach to the surface of dental materials. The polymer matrix, in resin-based materials like resin composites and RMGI, might absorb nicotine molecules more effectively than other substances do and cause color variations^27^. This corresponds with the results of Alandia-Roman et al.^31^ which show that the discoloration of resin composites becomes more noticeable as the concentration of staining agents, like nicotine, increases because of the way the material’s organic components interact with these substances.

Finally, the findings from this study support that exposure to nicotine can affect how well dental materials maintain their vulnerability to staining or discoloration over time – enamel stands up better against discoloration compared to RMGI, which is most prone to it, followed by resin composite materials.

### Limitations

The limitations of this study include the restricted range of e-cigarette nicotine concentrations examined (3, 20, and 50 mg), which may not fully represent the variety of nicotine levels commercially sold. Additionally, the exposure duration of 200 puffs may not accurately reflect the long-term effects on enamel and restorative materials. Furthermore, the study was conducted in a controlled laboratory setting using extracted teeth samples, which may limit its applicability to real-world clinical conditions. Potential residual confounding factors, such as variations in e-liquid composition and temperature fluctuations during aerosolization, were not accounted for and could influence the outcomes.

## CONCLUSIONS

The higher nicotine concentrations showed a greater effect among all samples in the tested groups. All concentrations of nicotine e-cigarettes (3, 20, and 50 mg) significantly affected the MH of all tested groups. In terms of SR, the only group that did not show a significant increase with all the e-cigarette nicotine concentrations is the RMGI. In aesthetic perspective, the lower the concentration of nicotine e-cigarettes, the lower the change in color when compared to higher concentrations. The significant color changes observed in RMGI and resin composites at higher nicotine concentrations highlight the need for clinicians to consider material choice when treating patients who use nicotine products, especially e-cigarettes. Future studies should incorporate longitudinal designs to evaluate cumulative exposure effects and assess interactions with other flavored e-cigarette formulations (e.g. menthol, fruit, or sweetened variants) and intraoral factors (e.g. saliva, temperature fluctuations), which may introduce additional chemical compounds affecting material degradation. Additionally, integrating real-world usage patterns (e.g. intermittent vs chronic vaping) and expanding nicotine concentration ranges (e.g. >50 mg) would further refine clinical guidelines for managing dental restorations in e-cigarette users.

## Data Availability

The data presented in this study are available on request from the corresponding author.

## References

[1] World Health Organization. Tobacco use declines despite tobacco industry efforts to jeopardize progress. WHO; 2024. Accessed March 11, 2025. https://www.who.int/news/item/16-01-2024-tobacco-use-declines-despite-tobacco-industry-efforts-to-jeopardize-progress

[2] O’Connor R, Schneller LM, Felicione NJ, Talhout R, Goniewicz ML, Ashley DL. Evolution of tobacco products: recent history and future directions. Tob Control. 2022;31(2):175-182. doi:10.1136/tobaccocontrol-2021-056544 35241585

[3] Etter JF. Electronic cigarettes: a survey of users. BMC Public Health. 2010;10:231. doi:10.1186/1471-2458-10-231 20441579 PMC2877672

[4] Hajek P, Etter JF, Benowitz N, Eissenberg T, McRobbie H. Electronic cigarettes: review of use, content, safety, effects on smokers and potential for harm and benefit. Addiction. 2014;109(11):1801-1810. doi:10.1111/add.12659 25078252 PMC4487785

[5] Dawkins L, Turner J, Roberts A, Soar K. ‘Vaping’ profiles and preferences: an online survey of electronic cigarette users. Addiction. 2013;108(6):1115-1125. doi:10.1111/add.12150 23551515

[6] Lindblom EN. Effectively regulating e-cigarettes and their advertising--and the first amendment. Food Drug Law J. 2015;70(1):55-92. 26292472

[7] Thomas SC, Xu F, Pushalkar S, et al. Electronic cigarette use promotes a unique periodontal microbiome. mBio. 2022;13(1):e0007522. doi:10.1128/mbio.00075-22 PMC8903898 35189698

[8] Pushalkar S, Paul B, Li Q, et al. Electronic cigarette aerosol modulates the oral microbiome and increases risk of infection. iScience. 2020;23(3):100884. doi:10.1016/j.isci.2020.100884 PMC7113564 32105635

[9] Sultan AS, Jessri M, Farah CS. Electronic nicotine delivery systems: oral health implications and oral cancer risk. J Oral Pathol Med. 2021;50(3):316-322. doi:10.1111/jop.12810 30507043

[10] Persoskie A, O’Brien EK, Poonai K. Perceived relative harm of using e-cigarettes predicts future product switching among US adult cigarette and e-cigarette dual users. Addiction. 2019;114(12):2197-2205. doi:10.1111/add.14730 31278802

[11] Kowitt SD, Meernik C, Baker HM, Osman A, Huang LL, Goldstein AO. Perceptions and experiences with flavored non-menthol tobacco products: a systematic review of qualitative studies. Int J Environ Res Public Health. 2017;14(4):338. doi:10.3390/ijerph14040338 28333107 PMC5409539

[12] Dalrymple A, Badrock TC, Terry A, et al. Development of a novel method to measure material surface staining by cigarette, e-cigarette or tobacco heating product aerosols. Heliyon. 2020;6(9):e05012. doi:10.1016/j.heliyon.2020.e05012 32995648 PMC7511806

[13] Alonazi M. Impact of smoking on resin bonded restorations: a narrative review. Tob Induc Dis. 2024;22:10.18332/tid/188114. doi:10.18332/tid/188114 PMC11135022 38813584

[14] Fagerstrom K. A Comparison of dependence across different types of nicotine containing products and coffee. Int J Environ Res Public Health. 2018;15(8):1609. doi:10.3390/ijerph15081609 30061507 PMC6121467

[15] Pintado-Palomino K, de Almeida CVVB, Oliveira-Santos C, Pires-de-Souza FP, Tirapelli C. The effect of electronic cigarettes on dental enamel color. J Esthet Restor Dent. 2019;31(2):160-165. doi:10.1111/jerd.12436 30367714

[16] Paolone G, Pavan F, Mandurino M, et al. Color stability of resin-based composites exposed to smoke. A systematic review. J Esthet Restor Dent. 2023;35(2):309-321. doi:10.1111/jerd.13009 36602255

[17] Alnasser HA, Elhejazi AA, Al-Abdulaziz AA, Alajlan SS, Habib SR. Effect of conventional and electronic cigarettes smoking on the color stability and translucency of tooth colored restorative materials: an in vitro analysis. Coatings. 2021; 11(12):1568. doi:10.3390/coatings11121568

[18] Ibrahim MS, Alatiyyah FM, Mohammed KA, Alhawaj HN, Balhaddad AA, Ibrahim AS. The effect of salbutamol and budesonide pediatric doses on dental enamel and packable and flowable composites: microhardness, surface roughness and color. Pharmaceutics. 2023;15(11):2527. doi:10.3390/pharmaceutics15112527 38004507 PMC10675679

[19] Ibrahim MS, Balhaddad AA, Garcia IM, et al. pH-responsive calcium and phosphate-ion releasing antibacterial sealants on carious enamel lesions in vitro. J Dent. 2020;97:103323. doi:10.1016/j.jdent.2020.103323 32360313

[20] Ibrahim MS, Alabbas MS, Alsomaly KU, et al. Flexural strength, elastic modulus and remineralizing abilities of bioactive resin-based dental sealants. Polymers (Basel). 2021;14(1):61. doi:10.3390/polym14010061 35012084 PMC8747332

[21] Benowitz NL, Fraiman JB. Cardiovascular effects of electronic cigarettes. Nat Rev Cardiol. 2017;14(8):447-456. doi:10.1038/nrcardio.2017.36 28332500 PMC5519136

[22] Soule E, Bansal-Travers M, Grana R, et al. Electronic cigarette use intensity measurement challenges and regulatory implications. Tob Control. 2023;32(1):124-129. doi:10.1136/tobaccocontrol-2021-056483 34059553 PMC8630087

[CIT0023] Bertoldo CEdS, Miranda DdA, Souza-Júnior EJ, et al. Surface hardness and color change of dental enamel exposed to cigarette smoke. Int J Dent Clin. 2011;3(4):1-4.

[24] Mathew M, Alanzi AL, Alruwaili TO. The effect of cigarette smoking on solubility and disintegration of resin modified glass ionomer cement–an in vitro study. J Dent Mater Technol. 2020;9(2):88–94. doi:10.22038/jdmt.2020.46287.1349

[25] Glantz SA. Heated tobacco products: the example of IQOS. Tob Control. 2018;27(Suppl 1):s1-s6. doi:10.1136/tobaccocontrol-2018-054601 30352841 PMC6252052

[26] Dorozhkin SV. Calcium orthophosphates: occurrence, properties, biomineralization, pathological calcification and biomimetic applications. Biomatter. 2011;1(2):121-164. doi:10.4161/biom.18790 23507744 PMC3549886

[27] Ahmed H. Craig’s Restorative Dental Materials. 14th ed. Br Dent J. 2019;226(9). doi:10.1038/sj.bdj.2019.29

[28] Yamamoto K, Ohashi S, Ando T, Watanabe M. Surface roughness and color stability of restorative materials exposed to various staining agents. Dent Mater J. 2018;37(1):123–30. doi:10.4012/dmj.2017-119.

[29] Giacomelli L, Derchi G, Frustaci A, et al. Surface roughness of commercial composites after different polishing protocols: an analysis with atomic force microscopy. Open Dent J. 2010;4:191-194. doi:10.2174/1874210601004010191 21228920 PMC3019617

[30] Karanjkar RR, Preshaw PM, Ellis JS, Holliday R. Effect of tobacco and nicotine in causing staining of dental hard tissues and dental materials: a systematic review and meta-analysis. Clin Exp Dent Res. 2023;9(1):150-164. doi:10.1002/cre2.683 36372903 PMC9932248

[31] Alandia-Roman CC, Cruvinel DR, Sousa AB, Pires-de-Souza FC, Panzeri H. Effect of cigarette smoke on color stability and surface roughness of dental composites. J Dent. 2013;41 Suppl 3:e73-e79. doi:10.1016/j.jdent.2012.12.004 23270748

